# Antiretroviral regimen switch and short-term viral suppression in persistent low-level viremia: A multicenter retrospective real-world cohort study in China

**DOI:** 10.1371/journal.pone.0355744

**Published:** 2026-08-21

**Authors:** Hao Liu, Yiming Ren, Lin Jia, Lijing Wang, Rugang Wang, Tongtong Yang, Yingquan Zhou, Hongxin Zhao, Lili Dai

**Affiliations:** 1 Center for Infectious Diseases, Beijing Youan Hospital, Capital Medical University, Beijing, China; 2 Department of Infectious Diseases, The Fifth Hospital of Shijiazhuang, Shijiazhuang, China; 3 Dalian Public Health Clinical Center, Dalian, China; 4 The Department of Infection, Chengdu Public Health Clinical Medical Center, Chengdu, China; 5 Department of Infectious Diseases, Lanzhou Pulmonary Hospital, Lanzhou, China; 6 Clinical Center for HIV/AIDS, Beijing Ditan Hospital, Capital Medical University, Beijing, China; Botswana-Harvard AIDS Research Institute, BOTSWANA

## Abstract

**Background:**

Persistent low-level viremia (pLLV) remains clinically challenging among people living with HIV (PWH) receiving antiretroviral therapy (ART), and whether ART regimen switching improves virologic outcomes remains controversial.

**Methods:**

We conducted a multicenter retrospective real-world cohort study across seven Chinese medical institutions. Adults with pLLV (50 copies/mL ≤ plasma viral load [VL] < 1000 copies/mL) were included and classified into a non-switch (control) group or a switch group based on the management decision of treating physicians at pLLV. The primary outcome was viral suppression at week 48; week 24 and week 96 suppression were secondary. Adjusted odds ratios (aOR) and risk differences (aRD) were estimated using logistic regression. Longitudinal viral load and CD4 trends were assessed using rank-based nonparametric longitudinal models.

**Results:**

162 participants were included (97 control; 65 switch). Viral suppression at week 48 was 64.37% in the control group and 68.52% in the switch group (aOR 1.40, P = 0.427; aRD 0.07, P = 0.423). At week 24, suppression occurred in 40.98% and 50.00%, respectively (aOR 1.34, P = 0.521; aRD 0.07, P = 0.518). HIV RNA levels decreased and CD4 increased modestly over time in both groups without between-group differences.

**Conclusions:**

In this retrospective cohort, regimen switching after pLLV was not associated with improved virologic suppression.

## Introduction

Antiretroviral therapy (ART) suppresses viral replication and reduces plasma viral load (VL) below assay detection limits in most people living with human immunodeficiency virus (PWH). Nevertheless, a subset of treated individuals may experience persistently detectable VL at low levels despite reported adherence, a phenomenon commonly termed persistent low-level viremia (pLLV) [1]. In China, the prevalence of pLLV has been estimated at approximately 2.6% [2]. Accumulating evidence links pLLV to unfavorable outcomes, including increased HIV reservoir complexity [3], impaired immune reconstitution and persistent inflammation [4–6], subsequent virologic failure [7–10], and an increased risk of non-AIDS events and mortality [11–14].

Definitions and management recommendations for pLLV differ across guidelines. The U.S. Department of Health and Human Services (DHHS) guidelines define LLV as two consecutive VL measurements above the lower limit of detection but <200 copies/mL [15]. The World Health Organization (WHO) guidelines use the operational range of detectable VL below the virologic failure threshold (<1000 copies/mL) [16]. Because VL is low, resistance testing in PWH with LLV is often unsuccessful, making it difficult to guide regimen modification according to resistance results. Therefore, guidelines recommend first addressing adherence and drug-drug interactions (DDIs), maintaining the current regimen, and repeating VL testing before considering regimen modification [15,16]. A randomized trial in Lesotho reported higher viral suppression among patients with pLLV switching from first-line regimen based on non-nucleoside reverse transcriptase inhibitor (NNRTI) to second-line regimen based on protease inhibitors (PI) [17]. Another study found that viremia events (VL ≥ 50 copies/mL) occurred in 10.4% of people receiving long-term integrase strand transfer inhibitor (INSTI)-based regimens, yet VL re-suppressed below the assay detection limit without any intervention, suggesting that regimen changes may not be necessary in such cases [18]. Overall, existing studies evaluating the effectiveness of regimen switching in pLLV have reported inconsistent results across settings and ART eras, and high-quality evidence from routine-care populations in China remains limited. Additional real-world studies are warranted to clarify optimal management strategies for pLLV in everyday clinical practice.

In this multicenter, retrospective, real-world study, we compared virologic outcomes between a regimen-switching strategy and a regimen-maintaining strategy among adults with pLLV. We assessed viral suppression at 24, 48, and 96 weeks, explored effect modification by baseline immune/virologic status and regimen category, and characterized longitudinal VL and CD4 trajectories to provide evidence relevant to clinical decision-making.

## Methods

### Study design and participants

This was a multicenter retrospective observational study. The data were obtained from the LLV database of seven medical institutions in China. As of December 31, 2025, we screened records of adults (≥18 years) with pLLV between June 1, 2018 and December 31, 2023 from the database for analysis. All data used in this study were fully anonymized prior to access by the investigators, and no identifiable personal information was available at any stage of the study. The investigators did not have access to any information that could identify individual PWH. pLLV was defined as two consecutive VL measurements in the range of 50 copies/mL ≤ VL < 1000 copies/mL, with the two measurements separated by at least 7 days. The inclusion criteria were receipt of ART for ≥6 months and documented pLLV. We excluded individuals who were pregnant or lactating, had documented ART interruption or withdrawal preceding the pLLV episode, had severe liver or kidney dysfunction, had severe opportunistic infections or malignancies at baseline, or lacked follow-up VL measurements within the prespecified windows. Participants were classified into the control group if they did not undergo regimen modification after pLLV confirmation, and into the switch group if ART was modified after pLLV confirmation during routine clinical care. The study protocol was approved by the Ethics Committee of Beijing Youan Hospital, Capital Medical University (2025131). Because of the retrospective design and the use of de-identified data, the requirement for informed consent was waived in accordance with the approved protocol.

The index date for follow-up was defined as the date of pLLV confirmation in the control group and the date of regimen switching in the switch group. Follow-up VL measurements were assessed at 24 weeks (6 months), 48 weeks (12 months), and 96 weeks (24 months) after the index date, using a ± 4-week visit window and selecting the measurement closest to the target visit when multiple measurements were available. Adherence counseling was strengthened for all patients with LLV; however, objective adherence measures, such as pharmacy refill records, pill counts, or validated adherence questionnaires, were not consistently available in the retrospective medical records and therefore could not be included as covariates in the analysis. VL and CD4 cell counts were abstracted at each time point when available; participants could contribute to analyses at a given time point if the relevant measurements were present, and they were considered missing otherwise.

### Outcomes

The primary outcome was the proportion of participants achieving viral suppression, defined as VL < 50 copies/mL, at week 48 after the index date. Secondary outcomes included viral suppression at weeks 24 and 96. Additional outcomes of interest were longitudinal trajectories of VL (log_10_-transformed) and CD4 cell counts, as well as laboratory measures used to describe safety (complete blood count and biochemical indices) at 12 and 24 months when available.

### Statistical methods

Assuming a two-sided test α of 0.05 and 80% power, and using effect sizes from a prior study [17](viral suppression 25% in the control group versus 55% in the switch group), the minimum sample size for comparing two independent proportions was 41 participants per group; accounting for 15% missingness, at least 49 participants per group were targeted. Categorical variables were presented as n (%) and were compared using χ² tests or Fisher’s exact tests as appropriate. Continuous variables were summarized as mean (standard deviations, SD), or median (interquartile ranges [IQR]) depending on distribution and were compared using Student’s t tests or Wilcoxon rank-sum tests. For binary outcomes, logistic regression models were fitted to estimate odds ratios (OR) and risk differences (RD) for switching versus maintenance. Effect estimates are reported with 95% confidence intervals (CIs). For continuous outcomes, we used linear regression models. Adjusted models included age, sex, baseline VL, baseline CD4 cell count, and current ART regimen category. No adjustment was made for medical centers, since some centers had few participants. Primary analyses were performed using available data at each time point. To assess robustness to missingness, we performed sensitivity analyses using multiple imputation by chained equations (MICE) to impute missing covariates and outcomes under a missing-at-random assumption.

To further address potential treatment-selection bias, we performed an inverse probability of treatment weighting (IPTW) sensitivity analysis. Propensity scores for regimen switching were estimated using a logistic regression model including age, sex, baseline HIV RNA log10, baseline CD4 count, current ART regimen class at pLLV confirmation, history of PI-based regimen, history of INSTI-based regimen, and drug resistance testing before ART. Stabilized weights were then calculated and applied to estimate weighted differences in virologic suppression between the switch and control groups. Covariate balance before and after weighting was assessed using absolute standardized mean differences (SMDs), with values <0.10 considered indicative of acceptable balance. Because baseline CD4 count was missing in some participants, IPTW was conducted as a complete-case sensitivity analysis. Center was not included in the propensity-score model because sparse center-level data produced unstable weights.

To explore potential heterogeneity of treatment effects, we conducted prespecified subgroup analyses by (1) current ART regimen category (NNRTI-, PI-, or INSTI-based), (2) baseline VL tertiles, and (3) baseline CD4 tertiles. Interaction was tested by adding a group×subgroup term to the logistic regression model and comparing models with and without the interaction term using likelihood ratio tests (P for interaction). Longitudinal VL and CD4 trends were assessed using rank-based nonparametric longitudinal models with a mixed between-within (F1-LD-F1) design; Wald-type statistics (WTS) and ANOVA-type statistics (ATS) were reported. All tests were two-sided, with P < 0.05 considered statistically significant. All statistical analyses were performed in R software (version 4.5.1) without adjustment for multiple testing. Multiple imputation was implemented with the mice package, and analyses of nonparametric longitudinal data were performed with the nparLD package.

## Results

**Fig 1:**
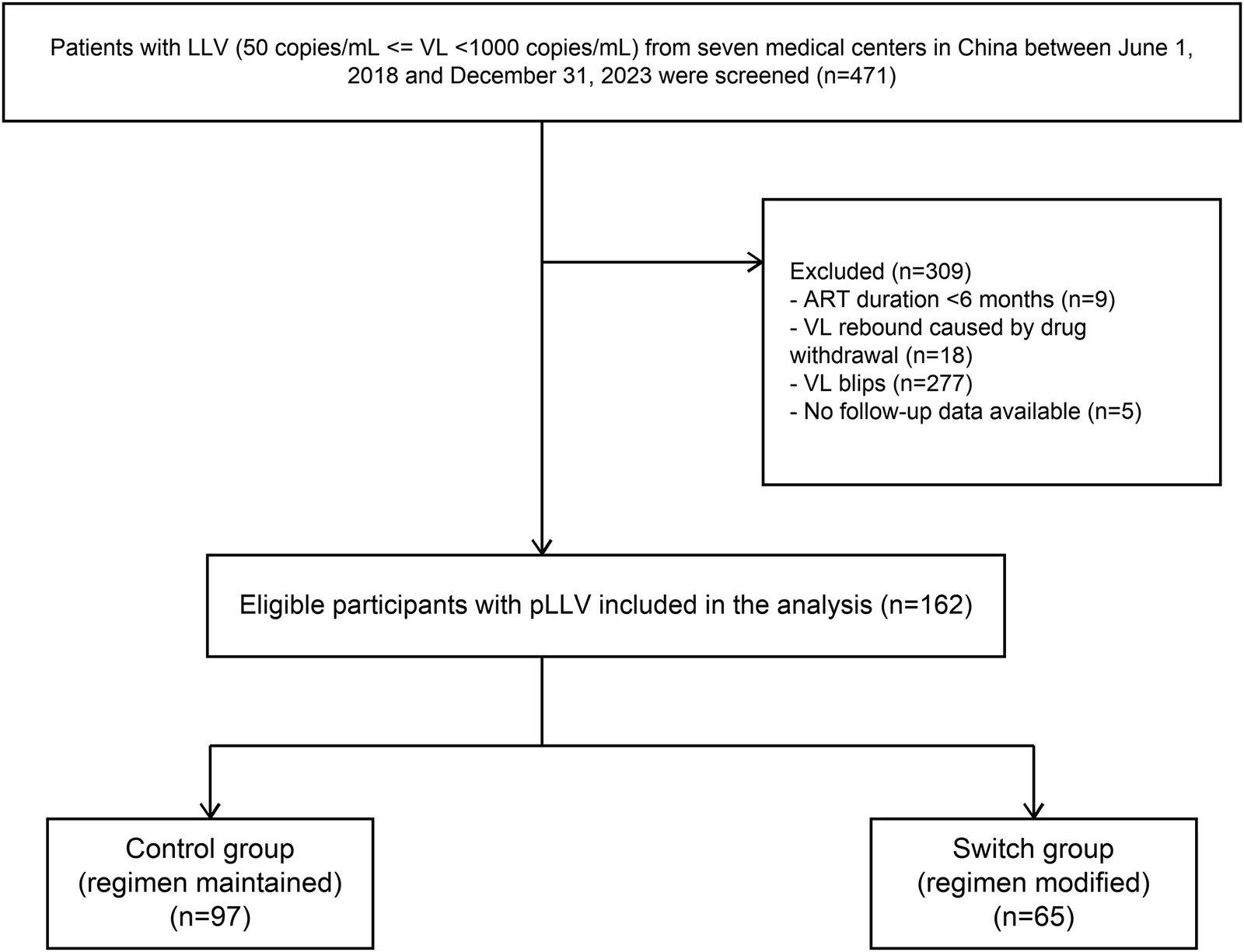
Flow diagram of participant screening and group allocation. LLV, low-level viremia; VL, viral load; ART, antiretroviral therapy.

From the electronic medical record databases of seven centers, adults with pLLV between June 1, 2018 and December 31, 2023 were screened for eligibility (Fig 1). After applying the predefined inclusion and exclusion criteria, 162 PWH with pLLV were included in the analysis. Among them, 97 were classified into the control group because they maintained their existing ART regimen after pLLV confirmation, whereas 65 were classified into the switch group because ART was modified during routine clinical care. Fig 1 summarizes the screening process, reasons for exclusion, and final allocation of participants into the two management groups.

In the control group, the median age (IQR) was 42 years (37–54), and 92 (94.80%) participants were male. In the switch group, the median age (IQR) was 48 years (40.00–57.00), and 60 (92.30%) participants were male. At the time pLLV was identified, 51 (52.60%) participants in the control group were receiving a NNRTI-based regimen, 12 (12.40%) a PI-based regimen, and 34 (35.1%) an INSTI-based regimen; the corresponding proportions in the switch group were 28 (43.10%), 10 (15.40%), and 27 (41.50%), respectively. Resistance testing after pLLV was more frequent in the switch group than in the control group (49.23% vs 42.27%; P = 0.019), and the switch group had a higher proportion of detected NRTI- and NNRTI-associated drug resistance mutations. Among the 65 participants in the switch group, the most common post-switch regimen class was INSTI-based therapy. Switch patterns included NNRTI-based to INSTI-based regimens in 22 participants (33.8%), PI-based to INSTI-based regimens in 9 (13.8%), and switching within INSTI-based regimens in 26 (40.0%). BIC/FTC/TAF was the most frequently used post-switch regimen (33/65, 50.8%). Detailed pre- and post-switch regimen patterns are shown in Tables A and B in S1 File. Additional baseline characteristics are presented in Table 1.

**Table 1:** Baseline characteristics of participants.

	Total (n = 162)	Control group (n = 97)	Switch group (n = 65)	*P*-value
Sex, male	152 (93.80%)	92 (94.80%)	60 (92.30%)	0.524
Age, year	43 [37.00;54.80]	42 [37.00;54.00]	48 [40.00;57.00]	0.102
^a^HBV infection	8 (5.00%)	5 (5.15%)	3 (4.76%)	>0.999
^b^HCV infection	3 (1.88%)	2 (2.06%)	1 (1.59%)	>0.999
Drug resistance testing before ART	57(35.19%)	34(35.05%)	23(35.38%)	>0.999
NRTI-associated DRM	4 (7.02%)	2 (5.88%)	2 (8.70%)	>0.999
NNRTI-associated DRM	10 (17.50%)	6 (17.60%)	4 (17.40%)	>0.999
PI-associated DRM	2 (3.51%)	2 (5.88%)	0 (0.00%)	0.51
INSTI-associated DRM	1 (1.75%)	1 (2.94%)	0 (0.00%)	>0.999
History of treatment^*^				>0.999
NNRTI-based	118 (72.80%)	71 (73.20%)	47 (72.30%)	
PI-based	34 (21.00%)	15 (15.50%)	19 (29.20%)	
INSTI-based	77 (47.50%)	37 (38.10%)	40 (61.50%)	
Combined with injectable ART	4 (2.47%)	1 (1.03%)	3 (4.62%)	
Current ART regimen at screening				0.493
NNRTI-based	79 (48.80%)	51 (52.60%)	28 (43.10%)	
PI-based	22 (13.60%)	12 (12.40%)	10 (15.40%)	
INSTI-based	61 (37.70%)	34 (35.10%)	27 (41.50%)	
HIV VL, copies/mL	132 [83.40;263.00]	120 [70.70;192.00]	169 [102.00;318.00]	0.004
HIV VLlog	2.12 [1.92;2.42]	2.08 [1.85;2.28]	2.23 [2.01;2.50]	0.004
^c^CD4 count, cells/mm^3^	391 [260.00;597.00]	399 [261.00;632.00]	389 [260.00;594.00]	0.913
^d^WBC, 10^9^/L	5.78 [4.99;7.17]	5.94 [4.89;7.10]	5.76 [5.15;7.47]	0.405
^e^HGB, g/L	156 [148.00;163.00]	156 [147.00;164.00]	154 [148.00;163.00]	0.666
^f^ALT, U/L	26 [19.00;39.40]	26 [18.00;37.00]	26 [22.00;40.20]	0.362
^g^AST, U/L	23 [18.60;31.80]	21 [17.60;29.20]	26 [20.00;33.80]	0.074
^h^eGFR, mL/min/1.73m²	108 [88.1;118.00]	110 [87.90;119.00]	105 [88.20;118.00]	0.625
^i^TG, mmol/L	1.97 [1.29;3.63]	1.77 [1.26;3.10]	2.26 [1.59;3.71]	0.141
^j^TC, mmol/L	4.84 (1.09)	4.84 (1.15)	4.85 (1.01)	0.935
^k^LDL, mmol/L	2.65 [2.10;3.19]	2.64 [2.07;3.23]	2.66 [2.11;3.05]	0.694
Drug resistance testing after LLV was detected	73(45.06%)	41(42.27%)	32(49.23%)	0.019
NRTI-associated DRM	8 (11.00%)	1 (2.44%)	7 (21.90%)	0.018
NNRTI-associated DRM	12 (16.40%)	3 (7.32%)	9 (28.10%)	0.039
PI-associated DRM	2 (2.74%)	1 (2.44%)	1 (3.12%)	>0.999
INSTI-associated DRM	0 (0.00%)	0 (0.00%)	0 (0.00%)	/
Change treatment				/
NNRTI-based	1 (0.62%)	/	1 (1.54%)	
PI-based	7 (4.32%)	/	7 (10.80%)	
INSTI-based	57 (35.20%)	/	57 (87.70%)	

Data are shown as n (% of PWH with nonmissing data) for categorical variables, median [IQR] for non-normally distributed continuous variables, and mean(±SD) for normally distributed continuous variables. Drug resistance mutation percentages were calculated among participants with available resistance testing results. HBV, hepatitis B virus; HCV, hepatitis C virus; ART, antiretroviral therapy; NRTI, nucleoside reverse transcriptase inhibitors; NNRTI, non-nucleoside reverse transcriptase inhibitors; PI, protease inhibitor; INSTI, integrase strand transfer inhibitors; DRMs, Drug Resistance Mutations; HIV, human immunodeficiency virus; VL, viral load; WBC, white blood cell; HGB, hemoglobin; ALT, alanine aminotransferase; AST, aspartate aminotransferase; eGFR, estimated glomerular filtration rate; TG, triglycerides; TC, total cholesterol; LDL, low-density lipoprotein; IQR, interquartile range; LLV, low-level viremia.

* All the treatment regimens used by the patients were recorded, and multiple options were allowed.

^a^Not measured for 2 participants in switch group.

^b^Not measured for 2 participants in switch group.

^c^Not measured for 10 participants in control group and 7 participants in switch group.

^d^Not measured for 2 participants in control group and 8 participants in switch group.

^e^Not measured for 2 participants in control group and 8 participants in switch group.

^f^Not measured for 1 participant in control group and 7 participants in switch group.

^g^Not measured for 1 participant in control group and 7 participants in switch group.

^h^Not measured for 13 participants in control group and 10 participants in switch group.

^i^Not measured for 16 participants in control group and 11 participants in switch group.

^j^Not measured for 16 participants in control group and 11 participants in switch group.

^k^Not measured for 16 participants in control group and 11 participants in switch group.

Because follow-up VL measurements were obtained from routine outpatient medical records rather than protocol-scheduled study visits, the availability of VL data varied across follow-up time points. VL measurements were available for 61/97 (62.9%) participants in the control group and 42/65 (64.6%) in the switch group at 24 weeks, for 87/97 (89.7%) and 54/65 (83.1%) at 48 weeks, and for 67/97 (69.1%) and 30/65 (46.2%) at 96 weeks, respectively (Table C in S1 File). The main documented reason for unavailable VL data was the absence of VL testing within the prespecified visit window. When baseline characteristics were compared according to the availability of week-48 VL measurements, participants without week-48 VL data had a lower frequency of drug resistance testing before ART and lower baseline eGFR, whereas most other baseline characteristics were broadly similar between groups (Table D in S1 File).

For the primary endpoint at week 48, VL data were available for 87/97 (89.69%) participants in the control group and 54/65 (83.08%) in the switch group (Table 2). Viral suppression was achieved in 56/87 (64.37%) and 37/54 (68.52%), respectively (adjusted OR 1.40, 95% CI 0.61–3.27; P = 0.427; adjusted RD 0.07, 95% CI −0.10 to 0.24; P = 0.423). For the secondary endpoint at week 24, suppression was achieved in 25/61 (40.98%) and 21/42 (50.00%), respectively (adjusted OR 1.34, 95% CI 0.55–3.28; P = 0.521; adjusted RD 0.07, 95% CI −0.14 to 0.27; P = 0.518). To further address potential treatment-selection bias, we performed a complete-case IPTW sensitivity analysis. In this analysis, 145 participants had complete covariate data for the propensity-score model. Stabilized weights were not extreme, and covariate balance improved after weighting, with all post-weighting absolute SMDs < 0.10 (Table E in S1 File). The IPTW results were consistent with the primary analysis. At 24 weeks, weighted viral suppression was 45.3% in the control group and 46.0% in the switch group (OR 1.03, 95% CI 0.44–2.41). At 48 weeks, weighted viral suppression was 63.4% and 64.0%, respectively (OR 1.02, 95% CI 0.48–2.18) (Table F in S1 File). Sensitivity analyses using MICE produced directionally similar effect estimates (Table G in S1 File). At week 96, outcome data were substantially incomplete; therefore, analyses at 96 weeks are presented as exploratory in Table H in S1 File and interpreted cautiously.

**Table 2:** Primary and secondary virologic outcomes after the index date.

	Overall (n = 162)	Control group (n = 97)	Switch group (n = 65)	Odds ratio (95% *CI*)	*P*-value	Risk difference (95% *CI*)	*P*-value
^**a**^ **Primary endpoint**							
VL < 50 copies/mL at 48 weeks	93 (65.96%)	56 (64.37%)	37 (68.52%)	1.40 (0.61, 3.27)	0.427	0.07 (−0.10, 0.24)	0.423
^**b**^ **Secondary endpoint**							
VL < 50 copies/mL at 24 weeks	46 (44.66%)	25 (40.98%)	21 (50.00%)	1.34 (0.55, 3.28)	0.521	0.07 (−0.14, 0.27)	0.518

Estimated by logistic regression adjusted for age, sex, VL, CD4 count and current ART regimen at screening. VL, viral load.

^a^Data were not available for 10 participants in control group and 11 participants in switch group.

^b^Data were not available for 36 participants in control group and 23 participants in switch group.

In prespecified subgroup analyses of week-48 viral suppression, effect estimates varied across strata of ART regimen, baseline VL tertiles, and baseline CD4 tertiles (Tables I and J, Figs A and B in S1 File). PWH already receiving an INSTI-based regimen did not appear to derive additional benefit from switching, whereas those with lower baseline CD4 counts showed a directionally more favorable estimate for switching; however, confidence intervals were wide and frequently crossed the null, reflecting limited precision. No subgroup-by-treatment interactions reached statistical significance in either unadjusted or adjusted interaction testing (all P for interaction >0.05). The test for interaction stratified according to ongoing regimen showed a borderline trend (unadjusted, 0.067; adjusted, 0.057) but did not meet the traditional threshold for significance.

**Fig 2:**
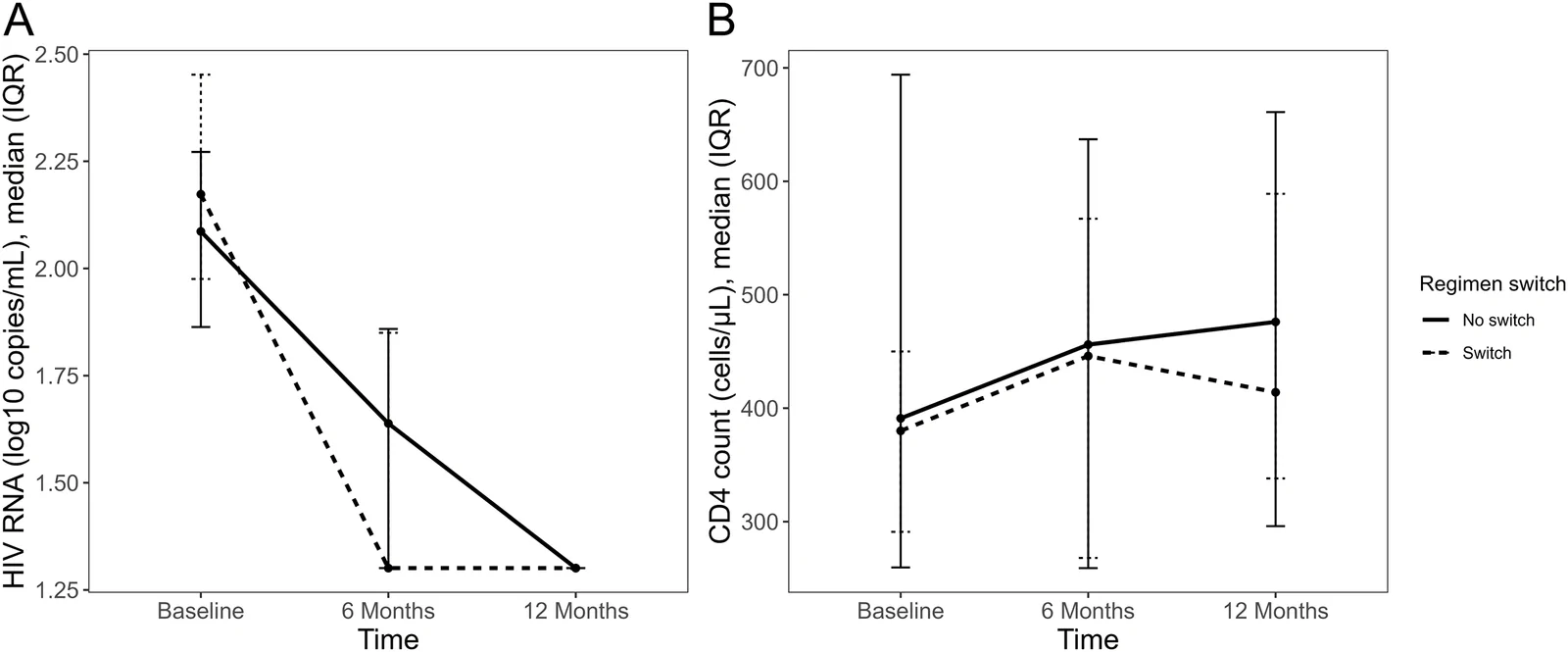
Longitudinal trends in HIV RNA and CD4 count during follow-up. (A) HIV RNA levels over time. (B) CD4 cell counts over time. Points indicate medians and error bars indicate interquartile ranges.

Fig 2 illustrates the longitudinal patterns of HIV RNA and CD4 count among PWH with complete measurements across the three follow-up time points. For HIV RNA, 54 participants in the control group and 38 in the switch group had complete longitudinal data (Fig 2A). HIV RNA levels decreased over time in both groups, with a significant main effect of time (WTS and ATS P < 0.001), but there was no significant main effect of group (P = 0.283) and no significant group-by-time interaction (WTS P = 0.167; ATS P = 0.155), indicating broadly similar VL trajectories between the two management strategies (Table K in S1 File). For CD4 count, complete longitudinal data were available for 37 control participants and 21 switch participants (Fig 2B). CD4 counts increased modestly over time, with a significant effect of time (WTS and ATS P < 0.01), but no significant group effect (P = 0.571) or group-by-time interaction (WTS P = 0.440; ATS P = 0.418), suggesting no statistically evident difference in CD4 recovery trajectories between groups (Table K in S1 File).

In exploratory analyses of safety-related laboratory measures, because of substantial missingness, we performed cross-sectional comparisons at 12 months and, for measures with available data, at 24 months (Table L in S1 File). Overall, distributions of white blood cell count, hemoglobin, liver enzymes, and lipid indices were similar between groups at both time points. Estimated glomerular filtration rate (eGFR) was higher in the control group at 12 months (P = 0.042), whereas no between-group difference was observed at 24 months (P = 0.155). Overall, we found no evidence that ART switching was associated with clinically meaningful adverse laboratory signals.

## Discussion

Whether and when to modify ART for pLLV remains a common dilemma in routine HIV care [19]. In this multicenter retrospective real-world cohort study, we addressed missing data using MICE, adjusted for prespecified confounders, and conducted subgroup analysis and interaction analyses to explore potential effect heterogeneity, thereby strengthening the robustness and credibility of our findings. We found that regimen switch after pLLV was not associated with a statistically significant improvement in viral suppression at 24 or 48 weeks, and longitudinal VL and CD4 trajectories were broadly similar between switching and maintaining strategies. Taken together, these findings do not support routine regimen change for PWH with pLLV in the absence of signals of virologic failure, and they reinforce the central role of adherence assessment and virologic monitoring in pLLV management, which is consistent with the current guidelines [15,16].

Prior studies have consistently identified lower CD4 cell counts and higher HIV RNA levels as risk factors for pLLV and subsequent virologic failure [2,20,21]. Compared with individuals who achieve viral suppression, those with LLV tend to have poorer CD4 cell count recovery during ongoing ART and a higher likelihood of CD4 decline to <200 cells/μL [6,22]. Together, these findings suggest a self-reinforcing cycle among individuals who enter pLLV with low baseline CD4 cell counts. In our subgroup analyses, the point estimates suggested that PWH in lower CD4 strata might derive greater benefit from switching, although interaction tests were not statistically significant and confidence intervals were wide. This pattern may reflect that immunocompromised individuals are more susceptible to ongoing low-level replication or less tolerant of modest pharmacologic shortcomings. Clinically, it suggests that regimen modification could be considered more selectively in PWH with advanced immunosuppression and pLLV, ideally informed by resistance testing and adherence evaluation. Larger studies are needed to define CD4 cell count thresholds that meaningfully influence prognosis among individuals with LLV and to inform regimen optimization for immunocompromised populations.

Previous studies in China have reported that drug resistance mutations are detected in >40% of individuals with LLV, with NNRTI- and NRTI-associated mutations each exceeding 20% [23,24]. Drug resistance is also associated with poorer immunologic outcomes, including a higher risk of very low CD4 cell counts [25]. Accordingly, resistance testing is important in the evaluation and management of LLV [26–28]. In our cohort, PWH in the switch group were more likely to undergo resistance testing and had more detected mutations. This pattern should be interpreted cautiously. More frequent testing may have increased the probability of detecting resistance mutations, thereby introducing detection bias. In addition, resistance testing and regimen switching were likely influenced by clinicians’ perceptions of virologic risk; PWH selected for switching may therefore have differed systematically from those managed without switching, consistent with confounding by indication. These factors may have biased the estimated association between regimen switching and virologic suppression. Nevertheless, regimen modification remains clinically appropriate for PWH with confirmed resistance mutations after comprehensive assessment. For PWH without available resistance testing, guidelines generally emphasize selecting regimens with a high genetic barrier, good tolerability, and minimized drug–drug interactions in suspected pLLV or in those at risk of virologic failure [15,16]. Prior studies suggest that switching from NNRTI-based therapy to PI-based therapy, or from NNRTI-/PI-based therapy to higher-barrier INSTI-based regimens, may facilitate re-suppression from LLV to undetectable viremia, while one study reported no significant difference between three-drug and two-drug regimens in this setting [17,29].

In our study, ART switch was not significantly associated with viral suppression in the primary analysis, which differs from the Lesotho trial [17]. This may be partly because, in our cohort, the control group was not exclusively treated with NNRTI-based regimens, and switching in the switch group did not uniformly represent an escalation to higher-genetic-barrier therapy. Specifically, approximately half of the control group was already receiving high-barrier PI- or INSTI-based regimens, and 40% of the switch group modified therapy within INSTI-based regimens. Subgroup analyses were directionally more favorable for switching among those receiving NNRTI- or PI-based therapy, whereas no clear benefit was observed among participants already on INSTI-based regimens, with only a borderline interaction trend by regimen category. These findings are consistent with prior evidence that most patients receiving bictegravir/emtricitabine/tenofovir alafenamide can re-suppress after viremia events without regimen changes [18]. A plausible interpretation is that, once a high-barrier regimen is in place, many pLLV episodes are driven less by ongoing replication under drug pressure and more by intermittent release from reservoirs or transient adherence fluctuations. In such circumstances, regimen changes may add complexity and potential toxicity without clear incremental benefit. Therefore, switching to higher-barrier therapy may be considered for patients on first-line regimens with a higher resistance risk after close monitoring and comprehensive evaluation, whereas management of LLV on high-barrier regimens should prioritize adherence, drug–drug interactions, and assay variability rather than premature regimen modification [30,31].

Mechanistically, pLLV is heterogeneous and may arise from at least two broad processes [32]: persistent replication under suboptimal drug exposure or incomplete adherence [33], and intermittent virus production from long-lived reservoirs without ongoing replication [34–37]. In the latter setting, changing ART may not alter virologic test results [38]. Several lines of evidence—including spontaneous re-suppression without regimen change and lack of viral evolution—support a substantial reservoir-driven component in many patients [30]. Accordingly, regimen switch may have limited impact on viremia and may divert attention from modifiable factors such as adherence, DDI, and monitoring practices. Conversely, when pLLV reflects ongoing replication under drug pressure, regimen optimization guided by resistance testing and adherence support may improve outcomes. Given the absence of a benefit of switching regimens in our primary analysis, we speculate that, in the routine-care pathway represented by our cohort, pLLV was not predominantly driven by persistent replication that is readily corrected by switching, or that this mechanism was not prevalent enough to yield detectable between-group differences. For reservoir-driven virus production, emerging evidence suggests that transcriptional regulators of the HIV promoter may represent potential therapeutic targets [39]. Future work combining longitudinal deep sequencing, adherence measurements, and reservoir or immune biomarkers is needed to distinguish mechanistic subtypes of pLLV and tailor management strategies accordingly.

Taken together, these findings support an individualized approach to pLLV management rather than routine regimen switching for all patients. In patients with low-level VL without an upward trend, no confirmed resistance mutations, stable CD4 counts, and current use of a high-genetic-barrier regimen such as an INSTI- or PI-based regimen, close monitoring, adherence support, and assessment of drug–drug interactions may be appropriate. By contrast, regimen optimization should be considered for patients with confirmed resistance mutations, increasing or repeatedly detectable VL, low or declining CD4 counts, or ongoing pLLV while receiving a lower-genetic-barrier regimen, particularly after adherence barriers and drug–drug interactions have been assessed. This framework is consistent with the heterogeneity of pLLV mechanisms and may help distinguish patients who can be monitored from those who may benefit from targeted regimen modification.

## Limitations

Our study has several limitations. First, because medical record databases were not designed for population-level surveillance and were not shareable across institutions, we could not estimate the incidence of pLLV among all treated patients in participating centers. Second, as a retrospective real-world study, confounding by indication is possible: clinicians may preferentially switch regimens in patients perceived to be at higher risk, and residual confounding may remain despite multivariable adjustment. In addition, the index date differed between groups by design, with follow-up anchored at pLLV confirmation in the control group and at regimen switching in the switch group; therefore, the findings should be interpreted as associations observed in routine care rather than as the causal effect of an immediate switching strategy. Third, follow-up VL testing was not protocol-scheduled and was incomplete within prespecified visit windows, particularly at 96 weeks, limiting longer-term inference and potentially introducing selection bias. Fourth, we lacked complete data on ART adherence, drug concentrations, HIV DNA reservoirs, and inflammatory biomarkers, which may help distinguish mechanistic subtypes of pLLV. Finally, the modest sample size limited precision in subgroup analyses, and our findings require validation in larger prospective studies.

## Conclusion

This multicenter retrospective real-world cohort study suggests that, among adults with pLLV, regimen switch was not associated with improved short-term viral suppression or distinct VL/CD4 trajectories compared with maintaining the existing regimen. In the absence of clear evidence of virologic failure, clinical management may prioritize adherence assessment and close monitoring to avoid unnecessary regimen changes. Future studies should clarify pLLV mechanisms and identify subgroups most likely to benefit from targeted regimen modification.

## Supporting information

S1 File Supplementary figures and tables. This file contains the supplementary tables and figures supporting the analyses reported in the manuscript.
(DOCX)

S1 Dataset Data underlying the study. This Excel file contains the de-identified data underlying the analyses reported in the manuscript.
(XLSX)
