# Supplementary material for: Antiretroviral regimen switch and short-term viral suppression in persistent low-level viremia: A multicenter retrospective real-world cohort study in China
Source: PLoS One. 2026 Aug 21;21(8):e0355744. doi: 10.1371/journal.pone.0355744 (PMC13496426; doi:10.1371/journal.pone.0355744)
Supplement: Supplementary file 1 [file pone.0355744.s001.docx]

**Supplementary figures and tables**

Table A. Regimen switch patterns by regimen class in the switch group.

| **Pre-switch regimen class** | **Post-switch regimen class** | **n (%)** | **Week-48 viral suppression, n/N (%)** |
| --- | --- | --- | --- |
| NNRTI-based | PI-based | 6 (9.2) | 4/6 (66.7) |
| NNRTI-based | INSTI-based | 22 (33.8) | 13/22 (59.1) |
| PI-based | PI-based | 1 (1.5) | NA |
| PI-based | INSTI-based | 9 (13.8) | 8/9 (88.9) |
| INSTI-based | NNRTI-based | 1 (1.5) | NA |
| INSTI-based | INSTI-based | 26 (40.0) | 12/17 (70.6) |

Values are n (%) unless otherwise indicated. Percentages were calculated among participants in the switch group. Week-48 viral suppression was calculated among participants with available week-48 VL measurements within the prespecified visit window after the index date. NA indicates that no week-48 VL measurement was available in that subgroup. INSTI, integrase strand transfer inhibitor; NNRTI, non-nucleoside reverse transcriptase inhibitor; PI, protease inhibitor; VL, viral load.

Table B. Specific post-switch regimens in the switch group.

| Post-switch regimen | n (%) | Week-48 viral suppression, n/N (%) |
| --- | --- | --- |
| BIC/FTC/TAF | 33 (50.8) | 21/27 (77.8) |
| EVG/c/FTC/TAF | 5 (7.7) | 3/5 (60.0) |
| AZT/3TC+LPV/r | 4 (6.2) | 3/3 (100.0) |
| DTG+3TC | 4 (6.2) | 2/2 (100.0) |
| AZT/3TC+DTG | 2 (3.1) | 2/2 (100.0) |
| TDF+3TC+DTG | 2 (3.1) | 0/2 (0.0) |
| TDF+3TC+LPV/r | 2 (3.1) | 0/2 (0.0) |
| TDF+3TC/DTG | 2 (3.1) | 0/2 (0.0) |
| other single-participant regimens | 11 (16.9) | 6/11 (54.5) |

Values are n (%) among participants in the switch group. Specific post-switch regimens are listed as recorded in the retrospective medical records. Regimens used in only one participant were grouped as “other single-participant regimens” in the table for readability; the full regimen list was retained in the analysis dataset. ART, antiretroviral therapy; INSTI, integrase strand transfer inhibitor; NNRTI, non-nucleoside reverse transcriptase inhibitor; PI, protease inhibitor.

Table C. Availability of follow-up viral load measurements within prespecified visit windows.

| **Follow-up time point** | **Group** | **Total,**  **n** | **VL available,**  **n (%)** | **VL unavailable, n (%)** | **Main documented reason for unavailability** |
| --- | --- | --- | --- | --- | --- |
| 24 weeks (6 months) | Control group | 97 | 61 (62.9) | 36 (37.1) | No VL test within prespecified window |
| 24 weeks (6 months) | Switch group | 65 | 42 (64.6) | 23 (35.4) | No VL test within prespecified window |
| 48 weeks (12 months) | Control group | 97 | 87 (89.7) | 10 (10.3) | No VL test within prespecified window |
| 48 weeks (12 months) | Switch group | 65 | 54 (83.1) | 11 (16.9) | No VL test within prespecified window |
| 96 weeks (24 months) | Control group | 97 | 67 (69.1) | 30 (30.9) | No VL test within prespecified window |
| 96 weeks (24 months) | Switch group | 65 | 30 (46.2) | 35 (53.8) | No VL test within prespecified window |

VL, viral load. Because this retrospective study was based on routine outpatient medical records, follow-up VL testing was not scheduled by the investigators. VL was considered unavailable when no VL measurement was recorded within the prespecified visit window.

Table D. Baseline characteristics according to availability of week-48 viral load measurements.

| Characteristic | VL available (n=141) | VL unavailable (n=21) | P-value |
| --- | --- | --- | --- |
| Switch group | 54 (38.3) | 11 (52.4) | 0.240 |
| Sex, male | 133 (94.3) | 19 (90.5) | 0.620 |
| Age, years | 44.00 [38.00; 55.00] | 48.00 [37.00; 58.00] | 0.907 |
| HBV infection | 6 (4.3) | 2 (9.5) | 0.282 |
| HCV infection | 3 (2.2) | 0 (0.0) | >0.999 |
| Drug resistance testing before ART | 54 (38.3) | 3 (14.3) | 0.048 |
| Drug resistance testing after pLLV | 68 (48.2) | 5 (23.8) | 0.058 |
| History of NNRTI-based regimen | 104 (73.8) | 14 (66.7) | 0.599 |
| History of PI-based regimen | 32 (22.7) | 2 (9.5) | 0.251 |
| History of INSTI-based regimen | 65 (46.1) | 12 (57.1) | 0.361 |
| History of injectable regimen | 4 (2.8) | 0 (0.0) | >0.999 |
| Current ART regimen: NNRTI-based | 69 (48.9) | 10 (47.6) | 0.419 |
| Current ART regimen: PI-based | 21 (14.9) | 1 (4.8) |  |
| Current ART regimen: INSTI-based | 51 (36.2) | 10 (47.6) |  |
| Baseline HIV RNA, copies/mL | 131.00 [79.00; 227.00] | 189.00 [94.90; 338.00] | 0.338 |
| Baseline HIV RNA, log10 copies/mL | 2.12 [1.90; 2.36] | 2.28 [1.98; 2.53] | 0.338 |
| Baseline CD4 count, cells/mm3 | 395.83 [262.00; 597.00] | 326.00 [241.93; 682.00] | 0.407 |
| Baseline WBC, 10^9/L | 5.80 [4.94; 7.15] | 5.73 [5.10; 7.47] | 0.525 |
| Baseline HGB, g/L | 155.00 [146.00; 163.00] | 162.00 [151.00; 164.00] | 0.050 |
| Baseline ALT, U/L | 26.00 [18.00; 36.00] | 29.35 [21.75; 46.00] | 0.216 |
| Baseline AST, U/L | 23.00 [18.00; 31.00] | 26.00 [19.80; 33.00] | 0.251 |
| Baseline eGFR, mL/min/1.73m2 | 109.10 [91.90; 119.30] | 90.70 [72.30; 106.20] | 0.012 |
| Baseline TG, mmol/L | 1.97 [1.33; 3.92] | 1.94 [1.06; 2.72] | 0.333 |
| Baseline TC, mmol/L | 4.84 [4.30; 5.50] | 4.77 [3.52; 5.00] | 0.066 |
| Baseline LDL, mmol/L | 2.66 [2.10; 3.22] | 2.53 [1.93; 2.94] | 0.523 |

Values are n (%) for categorical variables and median [IQR] for continuous variables. Week-48 VL was considered unavailable when no VL measurement was recorded within the prespecified 48-week visit window. Percentages were calculated among participants with nonmissing data for each variable. P values were calculated using Fisher exact tests for categorical variables and Wilcoxon rank-sum tests for continuous variables. ART, antiretroviral therapy; HBV, hepatitis B virus; HCV, hepatitis C virus; INSTI, integrase strand transfer inhibitor; IQR, interquartile range; NNRTI, non-nucleoside reverse transcriptase inhibitor; PI, protease inhibitor; VL, viral load; WBC, white blood cell; HGB, hemoglobin; ALT, alanine aminotransferase; AST, aspartate aminotransferase; eGFR, estimated glomerular filtration rate; TG, triglyceride; TC, total cholesterol; LDL, low-density lipoprotein.

Table E. Covariate balance before and after inverse probability of treatment weighting.

| Covariate | SMD before weighting | SMD after weighting |
| --- | --- | --- |
| Age | 0.108 | 0.029 |
| Sex, male | 0.025 | 0.053 |
| Baseline HIV RNA, log10 copies/mL | 0.371 | 0.097 |
| Baseline CD4 count | 0.004 | 0.077 |
| History of PI-based regimen | 0.324 | 0.014 |
| History of INSTI-based regimen | 0.61 | 0.009 |
| Drug resistance testing before ART | 0 | 0.001 |
| Current NNRTI-based regimen | 0.255 | 0.038 |
| Current PI-based regimen | 0.095 | 0.025 |
| Current INSTI-based regimen | 0.187 | 0.021 |

IPTW, inverse probability of treatment weighting; SMD, standardized mean difference; ART, antiretroviral therapy; INSTI, integrase strand transfer inhibitor; NNRTI, non-nucleoside reverse transcriptase inhibitor; PI, protease inhibitor. IPTW was performed as a complete-case sensitivity analysis among participants with complete covariate data for the propensity-score model. Stabilized weights were estimated using age, sex, baseline HIV RNA log10, baseline CD4 count, current ART regimen class, history of PI-based regimen, history of INSTI-based regimen, and drug resistance testing before ART. Covariate balance was assessed using absolute SMDs; values <0.10 were considered indicative of acceptable balance.

Table F. IPTW sensitivity analysis of virologic suppression at 24 and 48 weeks.

| Outcome | N control | N switch | Weighted suppression control | Weighted suppression switch | Risk difference | Odds ratio (95% CI) |
| --- | --- | --- | --- | --- | --- | --- |
| 24 weeks (6 months) | 52 | 39 | 45.3% | 46.0% | 0.01 | 1.03 (0.44-2.41) |
| 48 weeks (12 months) | 79 | 47 | 63.4% | 64.0% | 0.01 | 1.02 (0.48-2.18) |

IPTW, inverse probability of treatment weighting; VL, viral load. Virologic suppression was defined as VL <50 copies/mL. The analysis was restricted to participants with complete covariate data for the propensity-score model and available VL measurements at each follow-up time point. Stabilized IPTW weights were derived from the same propensity-score model used for covariate balancing. Weighted suppression proportions are shown for each group. Risk differences represent the weighted absolute difference between the switch and control groups. Odds ratios were estimated using weighted logistic regression.

Table G. Sensitivity analysis of virologic outcomes at 6 months and 12 months of follow-up.

|  | **Odds ratio (95% *CI*)** | ***P*-value** | **Risk difference (95% *CI*)** | ***P*-value** |
| --- | --- | --- | --- | --- |
| VL＜50 copies/mL at 48 weeks | 1.32 (0.19,9.40) | 0.478 | 0.06 (0.01,0.10) | 0.016 |
| VL＜50 copies/mL at 24 weeks | 1.62 (0.22,11.73) | 0.319 | 0.10 (-0.03,0.23) | 0.131 |

Table S8. Virologic outcomes at 24 months of follow-up.

|  | **Overall**  **(n=162)** | **Control group (n=97)** | **Switch group (n=65)** | **Odds ratio**  **(95% *CI*)** | ***P*-value** | **Risk difference (95% *CI*)** | ***P*-value** |
| --- | --- | --- | --- | --- | --- | --- | --- |
| **unadjusted** | | | | | | | |
| VL＜50 copies/mL at 96 weeks | 72 (74.23%) | 52 (77.61%) | 20 (66.67%) | 0.50 (0.54,1.84) | 0.213 | -0.06 (-0.24,0.11) | 0.497 |
| **adjusted for age, sex, VL, CD4 count and current ART regimen at screening** | | | | | | | |
| VL＜50 copies/mL at 96 weeks | 72 (74.23%) | 52 (77.61%) | 20 (66.67%) | 0.68 (0.00,2.14) | 0.500 | -0.17 (-0.42,0.08) | 0.190 |

Estimated by logistic regression. VL, viral load.

Data were not available for 30 participants in control group and 35 participants in switch group.

Table H. Subgroup analyses regarding virologic outcomes (unajusted).

| **subgroup_level** | **Overall (n)** | **Control group (n)** | **Switch group (n)** | **Odds ratio (95% *CI*)** | ***P*-value** | ***P for interaction*** |
| --- | --- | --- | --- | --- | --- | --- |
| NNRTIbased | 69 | 41 | 28 | 1.47 (0.56,3.90) | 0.437 | 0.067 |
| PIbased | 21 | 12 | 9 | 8.00 (0.75,85.31) | 0.085 |  |
| INSTIbased | 51 | 34 | 17 | 0.41 (0.10,1.70) | 0.220 |  |
| VL low tertile | 49 | 36 | 13 | 1.12 (0.29,4.41) | 0.866 | 0.966 |
| VL middle tertile | 48 | 30 | 18 | 1.16 (0.34,3.96) | 0.815 |  |
| VL high tertile | 44 | 21 | 23 | 1.41 (0.40,4.91) | 0.593 |  |
| CD4 low tertile | 41 | 27 | 14 | 4.12 (0.77,22.18) | 0.099 | 0.277 |
| CD4 middle tertile | 44 | 28 | 16 | 0.96 (0.28,3.33) | 0.954 |  |
| CD4 high tertile | 41 | 24 | 17 | 0.92 (0.25,3.39) | 0.896 |  |

NRTI, nucleoside reverse transcriptase inhibitors; NNRTI, non-nucleoside reverse transcriptase inhibitors; PI, protease inhibitor; INSTI, Integrase strand transfer inhibitors; VL, viral load.

Fig A. Subgroup analyses regarding virologic outcomes (unajusted).

NRTI, nucleoside reverse transcriptase inhibitors; NNRTI, non-nucleoside reverse transcriptase inhibitors; PI, protease inhibitor; INSTI, Integrase strand transfer inhibitors; VL, viral load.

Table I. Subgroup analyses regarding virologic outcomes (ajusted).

| **subgroup_level** | **Overall (n)** | **Control group (n)** | **Switch group (n)** | **Odds ratio (95% *CI*)** | ***P*-value** | ***P for interaction*** |
| --- | --- | --- | --- | --- | --- | --- |
| NNRTIbased | 69 | 41 | 28 | 2.21 (0.66,7.43) | 0.200 | 0.057 |
| PIbased | 21 | 12 | 9 | 9.26 (0.50,172.53) | 0.136 |  |
| INSTIbased | 51 | 34 | 17 | 0.36 (0.08,1.67) | 0.192 |  |
| VL low tertile | 49 | 36 | 13 | 1.54 (0.30,8.01) | 0.603 | 0.633 |
| VL middle tertile | 48 | 30 | 18 | 0.51 (0.10,2.48) | 0.401 |  |
| VL high tertile | 44 | 21 | 23 | 1.96 (0.43,8.84) | 0.383 |  |
| CD4 low tertile | 41 | 27 | 14 | NA | NA | 0.229 |
| CD4 middle tertile | 44 | 28 | 16 | 0.66 (0.12,3.67) | 0.634 |  |
| CD4 high tertile | 41 | 24 | 17 | 0.90 (0.18,4.64) | 0.902 |  |

Estimated by logistic regression adjusted for age, sex, VL, CD4 count and current ART regimen at screening.

NRTI, nucleoside reverse transcriptase inhibitors; NNRTI, non-nucleoside reverse transcriptase inhibitors; PI, protease inhibitor; INSTI, Integrase strand transfer inhibitors; VL, viral load.

Fig B. Subgroup analyses regarding virologic outcomes (ajusted).

Estimated by logistic regression adjusted for age, sex, VL, CD4 count and current ART regimen at screening.

NRTI, nucleoside reverse transcriptase inhibitors; NNRTI, non-nucleoside reverse transcriptase inhibitors; PI, protease inhibitor; INSTI, Integrase strand transfer inhibitors; VL, viral load.

Table J. Nonparametric longitudinal analysis (nparLD, F1-LD-F1) for HIV RNA (log10) and CD4 count.

Outcome: HIV RNA_log10（n=92；Control =54，Switch=38；3 time points）

| **Effect** | **WTS Statistic** | ***P*-value** | **ATS Statistic** | ***P*-value** |
| --- | --- | --- | --- | --- |
| group | 1.154 | 0.283 | 1.154 | 0.283 |
| time | 293.065 | <0.001 | 140.535 | <0.001 |
| group × time | 3.581 | 0.167 | 1.870 | 0.155 |

Outcome: CD4（n=58；No switch=37，Switch=21；3 time points）

| **Effect** | **WTS Statistic** | ***P*-value** | **ATS Statistic** | ***P*-value** |
| --- | --- | --- | --- | --- |
| group | 0.322 | 0.571 | 0.322 | 0.571 |
| time | 11.151 | 0.0038 | 5.017 | 0.0094 |
| group × time | 1.641 | 0.440 | 0.838 | 0.418 |

Table K. Clinical and biomedical characteristics at 6 months, 12months and 24 months of follow-up.

|  | **Total (n=162)** | **Control group (n=97)** | **Switch group (n=65)** | ***P*-value** |
| --- | --- | --- | --- | --- |
| **6 months after treatment change** | | | | |
| HIV VL, copies/mL | 50.00 [20.00;87.00] | 50.00 [20.00;89.50] | 35.10 [20.00;78.20] | 0.534 |
| HIV VLlog | 1.70 [1.30;1.94] | 1.70 [1.30;1.95] | 1.50 [1.30;1.89] | 0.534 |
| Virological suppression | 46 (44.70%) | 25 (41.00%) | 21 (50.00%) | 0.482 |
| CD4 count, cells/mm^3^ | 453 [270.00;651.00] | 472 [284.00;659.00] | 434 [242.00;651.00] | 0.486 |
| **12 months after treatment change** | | | | |
| HIV VL, copies/mL | 20 [20.00;60.00] | 20 [20.00;62.90] | 20 [20.00;50.00] | 0.457 |
| HIV VLlog | 1.30 [1.30;1.78] | 1.30 [1.30;1.80] | 1.30 [1.30;1.70] | 0.457 |
| Virological suppression | 93 (66.00%) | 56 (64.40%) | 37 (68.50%) | 0.747 |
| CD4 count, cells/mm^3^ | 476 [321.00;665.00] | 492 [317.00;698.00] | 436 [334.00;642.00] | 0.972 |
| WBC, 10^9^/L | 5.99 [5.02;7.17] | 5.99 [5.08;7.28] | 5.86 [5.02;7.00] | 0.815 |
| HGB, g/L | 155 [146.00;162.00] | 155 [146.00;161.00] | 156 [146.00;164.00] | 0.497 |
| ALT, U/L | 24 [19.00;34.80] | 23 [18.00;33.00] | 25 [19.50;37.00] | 0.341 |
| AST, U/L | 23.50 [19.80;28.20] | 23.00 [19.00;27.00] | 24.00 [20.00;28.50] | 0.583 |
| eGFR, mL/min/1.73m² | 104 [88.50;117.00] | 111 [91.90;119.00] | 99.0 [87.30;113.00] | 0.042 |
| TG, mmol/L | 2.21 [1.31;3.32] | 2.29 [1.48;3.80] | 2.10 [1.17;3.18] | 0.256 |
| TC, mmol/L | 4.73 [4.24;5.33] | 4.73 [4.16;5.45] | 4.71 [4.30;5.31] | 0.597 |
| LDL, mmol/L | 2.73 (0.71) | 2.71 (0.75) | 2.75 (0.67) | 0.758 |
| **24 months after treatment change** | | | | |
| HIV VL, copies/mL | 20.0 [20.0;41.0] | 20.0 [20.0;20.0] | 20.0 [20.0;128] | 0.149 |
| HIV VLlog | 1.30 [1.30;1.61] | 1.30 [1.30;1.30] | 1.30 [1.30;2.08] | 0.149 |
| Virological suppression | 72 (74.20%) | 52 (77.60%) | 20 (66.70%) | 0.375 |
| CD4 count, cells/mm^3^ | 514 [342.00;730.00] | 520 [336.00;716.00] | 508 [364.00;816.00] | 0.342 |
| WBC, 10^9^/L | 5.92 [5.24.00;6.78.00] | 6.17 [5.23;6.99] | 5.76 [5.45;6.22] | 0.342 |
| HGB, g/L | 156 [149.00;164.00] | 155 [150.00;163.00] | 157 [149.00;166.00] | 0.705 |
| ALT, U/L | 27.00 [19.20;34.80] | 27.00 [21.00;36.00] | 25.40 [19.00;32.00] | 0.429 |
| AST, U/L | 23 [19.00;30.80] | 23 [18.00;31.00] | 25 [21.00;30.00] | 0.418 |
| eGFR, mL/min/1.73m² | 104 [87.80;115.00] | 107 [92.60;118.00] | 100 [85.90;110.00] | 0.155 |
| TG, mmol/L | 2.16 [1.40;4.35] | 2.55 [1.41;4.70] | 1.94 [1.40;3.81] | 0.806 |
| TC, mmol/L | 5.07 (0.96) | 5.10 (1.05) | 5.03 (0.81) | 0.731 |
| LDL, mmol/L | 2.88 [2.29;3.38] | 2.78 [2.20;3.32] | 3.10 [2.53;3.38] | 0.223 |

Values are calculated among participants with available measurements at each time point; denominators may vary across variables. Results are number (% of those with nonmissing data) for categorical variables, median[IQR] for non-normally distributed continuous variables, and mean(±SD) for normally distributed continuous variables. HIV, human immunodeficiency virus; VL, viral load; WBC, white blood cell; HGB, hemoglobin; ALT, alanine aminotransferase; AST, aspartate aminotransferase; eGFR, estimated glomerular filtration rate; TG, triglycerides; TC, total cholesterol; LDL, low-density lipoprotein; IQR, interquartile range.
